# Comparative survival outcomes of minimally invasive versus open radical nephroureterectomy for upper tract urothelial carcinoma in Taiwan

**DOI:** 10.1007/s00345-025-05829-5

**Published:** 2025-07-30

**Authors:** I-Hsuan Alan Chen, Wei-Ming Li, Hung-Lung Ke, Yi-Huei Chang, Chao-Hsiang Chang, Yao-Chou Tsai, Chih-Chin Yu, Wun-Rong Lin, Marcelo Chen, Yi-Hsin Lu, Chia-Cheng Yu

**Affiliations:** 1 Division of Urology, Department of Surgery, Kaohsiung Veterans General Hospital, 386 Ta-Chung 1st Rd., Zuoying Dist, Kaohsiung, Taiwan; 2 School of Medicine, College of Medicine, National Yang Ming Chiao Tung University, Taipei, Taiwan; 3 Division of Urology, Department of Surgery, Tri-Service General Hospital, National Defense Medical Center, Taipei, Taiwan; 4 Department of Urology, Kaohsiung Medical University Gangshan Hospital, Kaohsiung, Taiwan; 5 Department of Urology, Kaohsiung Medical University Hospital, Kaohsiung, Taiwan; 6 Department of Urology, School of Medicine, College of Medicine, Kaohsiung Medical University, Kaohsiung, Taiwan; 7 Graduate Institute of Medicine, College of Medicine, Kaohsiung Medical University, Kaohsiung, Taiwan; 8 Department of Urology, China Medical University Hospital, Taichung, Taiwan; 9 School of Medicine, China Medical University, Taichung, Taiwan; 10 Division of Urology, Department of Surgery, The Buddhist Tzu Chi Medical Foundation, Taipei Tzu Chi Hospital, New Taipei City, Taiwan; 11 School of Medicine, Buddhist Tzu Chi University, Hualien, Taiwan; 12 Department of Urology, Taipei Medical University Hospital, Taipei Medical University, Taipei City, Taiwan; 13 Department of Urology, MacKay Memorial Hospital, Taipei, Taiwan; 14 Mackay Medical College, Taipei, Taiwan; 15 Nursing, and Management, Mackay Junior College of Medicine, Taipei, Taiwan

**Keywords:** Minimally invasive surgery, Nephroureterectomy, Urothelial carcinoma

## Abstract

**Purpose:**

Upper tract urothelial carcinoma (UTUC) is rare globally but accounts for 30–40% of urothelial cancers in Taiwan. Radical nephroureterectomy (RNU) with bladder cuff excision (BCE) remains the standard of care for localized or locally advanced disease. Despite the increasing adoption of minimally invasive surgical (MIS) approaches, concerns about their oncological outcomes persist. This study evaluates the comparative survival outcomes of MIS versus open RNU for UTUC using propensity-score-matched (PSM) analysis.

**Methods:**

Data from 2430 patients with UTUC, treated between 1988 and 2022 within the Taiwan UTUC Collaboration Group, were retrospectively analyzed. PSM was employed to minimize baseline differences. The primary endpoints were overall survival (OS), cancer-specific survival (CSS), and disease-free survival (DFS). Kaplan-Meier estimates, stratified log-rank tests, and Cox proportional hazards models were used to evaluate survival outcomes.

**Results:**

After PSM, 1758 patients (1172 MIS; 586 open) were included. The MIS group demonstrated significantly improved OS (OR: 0.662; *p* < 0.001), CSS (OR: 0.659; *p* = 0.002), and DFS (OR: 0.646; *p* < 0.001) compared to the open group. MIS was associated with superior survival despite a higher prevalence of high-grade tumors and adverse pathological features.

**Conclusion:**

MIS approaches, including laparoscopic and robotic RNU, offer oncological outcomes comparable to or better than open RNU in UTUC. These findings support the broader adoption of MIS techniques, emphasizing meticulous BCE to ensure oncological control.

**Supplementary Information:**

The online version contains supplementary material available at 10.1007/s00345-025-05829-5.

## Introduction

Upper tract urothelial carcinoma (UTUC) is relatively rare worldwide; however, it represents a significant proportion of urothelial cancers in Taiwan, accounting for approximately 30–40% of cases [1]. Radical nephroureterectomy (RNU) with bladder cuff excision (BCE) remains the established gold standard for managing localized or locally advanced UTUC. Since the introduction of laparoscopic RNU in 1991 [2] and robotic RNU in 2007 [3], minimally invasive surgical (MIS) approaches have been widely adopted, revolutionizing the management of UTUC over the past two decades.

The benefits of minimally invasive surgery, including reduced intraoperative blood loss, shorter hospital stays, decreased perioperative morbidity, and faster postoperative recovery, are well-documented. However, debate persists regarding the oncological outcomes of MIS compared to traditional open surgery. A systematic review by the European Association of Urology suggests that conventional open RNU [4] offers superior oncological control compared to laparoscopic RNU, particularly in high-risk cases of locally advanced UTUC where laparoscopic bladder cuff excision is performed [5]. Conversely, a meta-analysis found no significant difference in oncological outcomes between open and laparoscopic RNU, even in cases of locally advanced disease [6]. Further support for MIS comes from a propensity-matched analysis of the National Cancer Database (NCDB), which reported improved overall survival (OS) in octogenarians undergoing MIS (both laparoscopic and robotic RNU) after a median follow-up of 39.4 months [4].

Within the Taiwanese UTUC Collaboration Group, 72% of patients treated between August 1988 and April 2021 underwent minimally invasive RNU, with a marked increase in MIS adoption observed after 2000 [7]. Multivariate analyses identified MIS approaches—specifically laparoscopic, hand-assisted laparoscopic, and robotic RNU—as favorable predictors of OS. Notably, laparoscopic RNU alone was found to be an independent predictor of reduced cancer-related mortality. To further investigate the potential survival advantages of MIS compared to conventional open procedures, we performed a propensity-score-matched (PSM) multivariable survival analysis, comparing OS and cancer-specific survival between MIS and open RNU.

This study aims to provide robust evidence comparing the survival outcomes of MIS and open RNU for UTUC, with the goal of informing surgical decision-making and enhancing the understanding of MIS’s role in oncological control for UTUC.

## Materials and methods

### Patient population

**Fig. 1:**
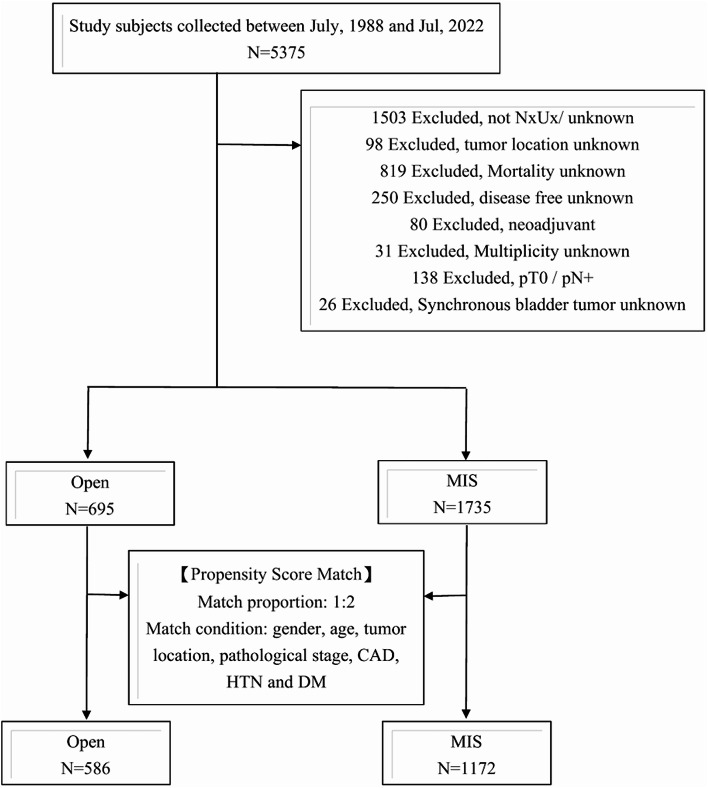
Study flowchart

This study, conducted by the Taiwan UTUC Collaboration Group, was reviewed and approved by the Kaohsiung Veterans General Hospital Institutional Review Board (IRB No.: VGHKS14-CT3-06). A total of 5375 patients with upper tract urothelial carcinoma (UTUC), enrolled between July 1988 and July 2022, were considered for inclusion. Of these, 2430 patients who underwent open or MIS RNU with bladder cuff excision were ultimately included in the analysis. The procedures were performed between August 9, 1988, and February 17, 2022. To ensure data completeness and analytical robustness, stringent exclusion criteria were applied (Fig. 1). Patients with incomplete data were excluded, and no imputation was performed, resulting in fully complete datasets for all extracted variables.

Patient demographics, postoperative complications, and their grading (based on the Clavien-Dindo classification) were recorded. Tumor location and size were determined from post-RNU specimen evaluations, with multifocality defined as the presence of two or more pathologically confirmed lesions at distinct anatomical sites (renal pelvis or ureter). Tumor size was measured as the sum of the longest diameters of all detected tumors. Preoperative hydronephrosis was assessed using computed tomography (CT) or magnetic resonance imaging (MRI). Pathological features—including tumor cell type, carcinoma in situ (CIS), lymphovascular invasion (LVI), tumor necrosis, and surgical margins—were reviewed by urological pathologists. Histological grading adhered to the 1973 and 2004 World Health Organization classification, while pathological staging was performed according to the 2017 AJCC TNM staging system. Additionally, the presence and timing of bladder UC were documented.

### Survival assessment

The primary endpoint of this study was to compare oncological outcomes between the open and MIS groups, specifically evaluating OS, cancer-specific survival (CSS), and disease-free survival (DFS). For the CSS analysis, patients who died within 30 days of RNU or during the same hospital stay were censored at the time of death. DFS was defined as the time from RNU to the first documented occurrence of local recurrence at the tumor bed, lymph node involvement, or distant metastasis, with recurrence and metastasis confirmed through radiological or pathological evaluation. Intravesical recurrence (IVR) was defined as any histologically confirmed bladder UC occurring at least 3 months post-RNU. All survival outcomes were analyzed using multivariate Cox proportional hazards models.

### Statistical method

Group differences were assessed using the Pearson Chi-square test for categorical variables, while normality of continuous variables was evaluated using the Student’s t-test. To address potential imbalances in confounding factors between the open and MIS RNU groups, propensity score matching was employed. Propensity scores were estimated using a logistic regression model, with surgical status as the dependent variable and baseline covariates—including gender, age, tumor location, pathological stage, coronary artery disease (CAD), hypertension, and diabetes mellitus (DM)—as predictors. Matched pairs were created between the groups using nearest-neighbor propensity-score matching, with tolerance levels set at 0.001 and 0.002.

Our study utilized one-to-two propensity score matching to form pairs of control and treated subjects with similar propensity scores. Prognostic outcome rates were estimated using the Kaplan-Meier method, and survival curves were compared with the stratified log-rank test. The effect of surgical approaches on prognostic outcomes was assessed using a Cox proportional hazards model, both unadjusted and adjusted for potential confounders. All statistical tests were two-tailed, with a significance threshold set at *p* < 0.05. Statistical analyses were conducted using IBM SPSS Statistics, version 26.

## Results

A total of 2430 patients were included in the study (Table 1). Of these, 1735 (71.4%) underwent MIS RNU, while 695 (28.6%) underwent open RNU. The age distribution was comparable between the two groups. Comorbidity analysis revealed a higher prevalence of CAD, arrhythmia, and DM among patients in the MIS group. There were no significant differences between groups regarding tumor location, multiplicity, pathological T staging, synchronous bladder tumors, or receipt of adjuvant treatment.

**Table 1:** Baseline demographic data of variables the UTUC patients

Variables	Overall					Propensity score matched				
	Open (*N* = 695		MIS (*N* = 1735		p value^a^	Open (*N* = 586		MIS (*N* = 1172		p value^a^
	N	%	N	%		N	%	N	%	
Gender										
Men	319	45.9	752	43.3	0.251	258	44.0	513	43.8	0.919
Women	376	54.1	983	56.7		328	56.0	659	56.2	
Age										
< 70	407	59.2	948	54.9	0.054	335	57.2	704	60.1	0.244
≥ 70	280	40.8	778	45.1		251	42.8	468	39.9	
Comorbidity										
CAD	26	3.8	175	10.2	< 0.001**	26	4.4	52	4.4	1.000
Arrythmia	266	38.8	944	55.2	< 0.001**	265	45.2	520	44.4	0.734
HTN	92	13.4	217	12.7	0.629	87	14.8	150	12.8	0.236
DM	127	18.5	458	26.8	< 0.001**	125	21.3	265	22.6	0.543
Malignancy (no UTUC/bladder UC)	74	10.8	208	12.2	0.348	69	11.8	155	13.2	0.390
Cell type										
Urothelial	646	92.9	1593	91.8	0.630	540	92.2	1070	91.3	0.698
UC with variants	44	6.3	129	7.4		41	7.0	94	8.0	
Others	5	0.7	13	0.7		5	0.9	8	0.7	
Laterality										
Left	350	50.6	878	50.6	0.990	299	51.0	602	51.4	0.893
Right	342	49.4	857	49.4		287	49.0	570	48.6	
Tumor location										
Renal pelvis	313	45.2	793	45.7	0.922	267	45.6	517	44.1	0.847
Ureter	249	35.9	626	36.1		212	36.2	435	37.1	
Renal pelvis + ureter	131	18.9	316	18.2		107	18.3	220	18.8	
Multifocality										
No	450	64.9	1114	64.6	0.869	383	65.4	748	64.2	0.634
Yes	243	35.1	611	35.4		203	34.6	417	35.8	
RNU histology										
Low grade	64	9.4	240	13.9	< 0.001**	58	9.9	141	12.1	< 0.001**
High grade	470	69.3	1444	83.5		410	70.2	997	85.3	
G2	144	21.2	45	2.6		116	19.9	31	2.7	
Pathological stage T										
pTis/pTa/pT1	299	45.3	787	45.6	0.081	268	45.7	533	45.5	0.116
pT2	143	21.7	356	20.6		125	21.3	234	(20.0	
pT3	186	28.2	538	31.2		168	28.7	376	32.1	
pT4	32	4.8	44	2.6		25	4.3	29	2.5	
Urinary bladder tumor										
No	501	72.2	1325	76.5	0.053	428	73.0	888	75.9	0.109
Previous history of bladder UC	58	8.4	107	6.2		50	8.5	69	5.9	
Concurrent Bladder UC	135	19.5	301	17.4		108	18.4	213	18.2	
CIS										
No	591	87.8	1331	77.3	< 0.001**	510	87.0	862	73.8	< 0.001**
Yes	82	12.2	390	22.7		76	13.0	306	26.2	
Lymphovascular invasion										
No	572	84.0	1387	81.7	0.191	488	83.4	940	82.4	0.590
Yes	109	16.0	310	18.3		97	16.6	201	17.6	
Surgical margin										
Free	637	96.5	1672	97.0	0.558	565	96.4	1137	97.0	0.501
Positive	23	3.5	52	3.0		21	3.6	35	3.0	
Tumor necrosis										
No	590	93.7	1403	82.9	< 0.001**	529	93.6	958	83.1	< 0.001**
Yes	40	6.3	289	17.1		36	6.4	195	16.9	
Adjuvant chemotherapy										
No	586	84.3	1440	83.0	0.430	496	84.6	955	81.5	0.100
Yes	109	15.7	295	17.0		90	15.4	217	18.5	
Adjuvant radiation therapy										
No	674	97.0	1682	96.9	0.966	569	97.1	1137	97.0	0.921
Yes	21	3.0	53	3.1		17	2.9	35	3.0	
RNU date										
Before the end of 2010	372	53.5	288	16.6	< 0.001**	287	49.0	139	11.9	< 0.001**
After the start of 2011	323	46.5	1447	83.4		299	51.0	1033	88.1	
Bladder-cuff excision										
Open incision	534	77.2	1069	63.5	< 0.001**	441	75.6	714	62.9	< 0.001**
Transurethral incision	157	22.7	150	8.9		141	24.2	110	9.7	
Laparoscopy	1	0.1	283	16.8		1	0.2	179	15.8	
Robot assisted	0	0.0	176	10.5		0	0.0	130	11.4	
LESS	0	0.0	5	0.3		0	0.0	3	0.3	
Clavien-Dindo classification										
No	499	73.1	1059	64.5	0.004**	415	71.7	690	63.0	0.008**
Grade I	57	8.3	182	11.1		51	8.8	129	11.8	
Grade II	107	15.7	331	20.1		93	16.1	232	21.2	
Grade III	9	1.3	40	2.4		9	1.6	28	2.6	
Grade IV	9	1.3	26	1.6		9	1.6	15	1.4	
Grade X	2	0.3	5	0.3		2	0.3	1	0.1	
Mortality										
No	280	40.3	1215	70.0	< 0.001**	256	43.7	880	75.1	< 0.001**
UTUC related	161	23.2	262	15.1		122	20.8	155	13.2	
Non-UTUC related	252	36.3	252	14.5		206	35.2	135	11.5	
Surgery related	2	0.3	6	0.3		2	0.3	2	0.2	
Disease free										
No	213	30.6	401	23.1	< 0.001**	168	28.7	254	21.7	0.001**
Yes	482	69.4	1334	76.9		418	71.3	918	78.3	
IVR after RNU										
No	494	71.1	1218	70.2	0.668	421	71.8	830	70.8	0.655
Yes	201	28.9	517	29.8		165	28.2	342	29.2	
Complication										
ESRD	60	8.8	186	10.9	0.132	54	9.3	108	9.3	0.983
Ileus	7	1.0	37	2.2	0.062	6	1.0	25	2.2	0.094
Ventral hernia	2	0.3	38	2.2	0.001**	2	0.3	22	1.9	0.009**
Follow up (months) ^c^	54.4	44.6	0.006**	56.1	41.6	< 0.001**				
Median (IQR)	22.8–103.7	25.6–76.3		24.3–102.4	24.5–70.7					

Following a median follow-up of 54.4 months, 1215 (70%) patients in the MIS group remained alive without disease recurrence, and 1334 (76.9%) were disease-free. In comparison, after a median follow-up of 44.6 months, 482 (69.4%) patients in the open RNU group were recurrence-free, and 280 (40.3%) were still alive. The use of MIS surged from 16.6% before the end of 2010 to 83.4% after the start of 2011, while the proportion of open RNU cases steadily declined, reaching 46.5% since 2011

^a^Chi-Squared test calculated for the difference Variables. ^b^ Student’s t-test calculated for the difference in means. ^c^ Wilcoxon rank-sum test calculated for the difference in medians. * < 0.05, ** < 0.01

### Propensity-score matching analysis

**Fig. 2:**
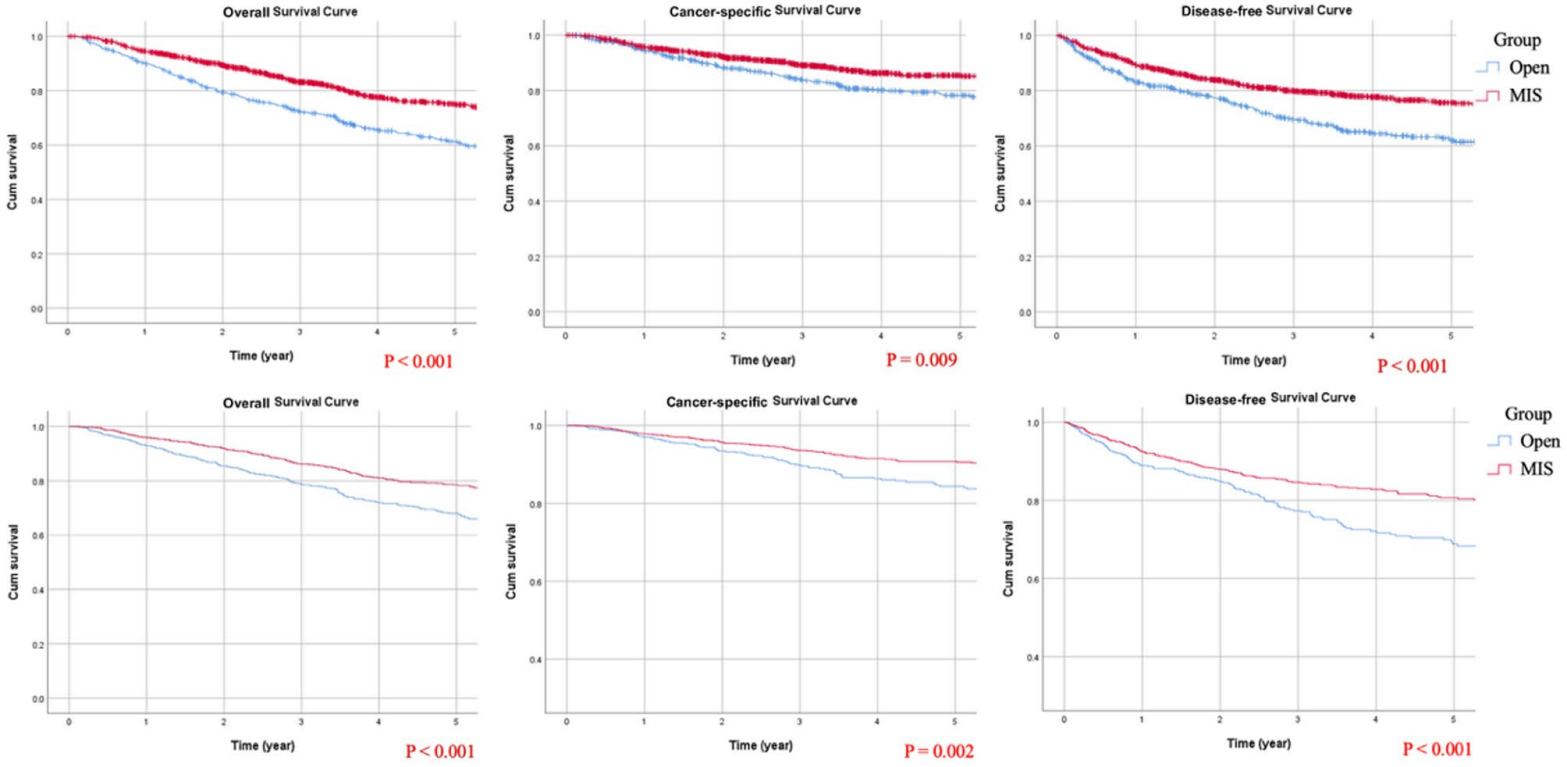
Kaplan–Meier survival curves: unadjusted (top row) and adjusted (bottom row) analyses for overall survival, cancer-specific survival, and disease-free survival

PSM was performed to compare MIS and open RNU cases, with each open RNU patient matched to two MIS patients. This resulted in a cohort of 1172 (66.7%) MIS patients and 586 (33.3%) open RNU patients. After matching, baseline characteristics—including gender, age, tumor location, pathological T stage, and comorbidities—were balanced between the two groups. Despite a higher proportion of patients with high-grade UC, concomitant CIS, and tumor necrosis in the MIS group, these patients demonstrated improved survival outcomes, including OS, CSS, and DFS. Adjusted Kaplan-Meier survival estimates are presented in Fig. 2. While MIS RNU was associated with improved survival outcomes, it was also linked to a higher incidence of postoperative morbidities requiring intervention.

### Univariable adjustment

In the PSM univariate analysis of OS, CSS, and DFS, significant favorable predictors included the MIS approach, age below 70, negative surgical margins, and surgical dates after January 2011. Unfavorable predictors included ureteral and multifocal UC, concurrent bladder UC, LVI, tumor necrosis, advanced pathological T staging, and receipt of adjuvant radiotherapy (Supplementary Table 1).

### Multivariable adjustment

The PSM-adjusted multivariable analysis revealed that patients undergoing MIS had significantly better survival outcomes compared to those undergoing open RNU, with longer OS (odds ratio [OR]: 0.662; *p* < 0.001), CSS (OR: 0.659; *p* = 0.002), and DFS (OR: 0.646; *p* < 0.001) (Supplementary Table 2. Adverse predictors of survival included ureteral UC, concurrent bladder UC, positive surgical margins, LVI, tumor necrosis, and advanced pathological T staging.

## Discussion

In the present study, the original cohort showed better OS, CSS, and DFS with the MIS approach for patients with UTUC. After balancing baseline characteristics, potential confounders—including sex, age, tumor location, pathologic T stage, and comorbidities—were comparable between the open and MIS groups. Despite a higher prevalence of adverse pathologic features (high-grade urothelial carcinoma, tumor necrosis, and carcinoma in situ) in the MIS group, PSM analysis still indicated that MIS may provide superior oncologic control compared with open RNU. Notably, both before and after PSM, a higher proportion of high-grade disease was presented in the MIS group. Multivariate analyses showed that tumor grade was not an independent predictor of overall survival, consistent with our previous findings [7].

Surgical margin status remains a critical prognostic factor. A multicenter database previously identified margin status as an independent predictor of metastasis-free survival after RNU [8]. Ditonno et al. recently reported a 4.7% positive margin rate among over 1100 patients in the ROBotic surgery for Upper tract Urothelial cancer STtudy (ROBUUST) 2.0 database who underwent robotic RNU [9]. In the current study, comparable positive margin rates were observed in the MIS and open groups (3.0% vs. 3.6%, respectively). Likewise, Rajan et al. found lower positive margin rates with robotic versus open or laparoscopic RNU in well-balanced studies [10], and Trecarten et al. linked MIS to reduced positive margin rates and lower 90-day mortality [4]. On the other hand, a meta-analysis by Veccia et al. showed that open RNU had the poorest 5-year CSS among 87,291 patients [11]. Despite limitations in the quality of evidence comparing surgical approaches for UTUC, an increasing body of data supports comparable or even superior survival outcomes with the MIS approach. A prior analysis comparing robotic and laparoscopic RNU was conducted based on the Taiwanese UTUC collaboration cohort [12]. Baseline characteristics were balanced using the overlap weighting method, and perioperative as well as survival outcomes were comparable between both groups.

Management of the distal ureter and bladder cuff is pivotal for oncologic control. Our study reflects contemporary Taiwanese practice, employing transurethral incision and open, laparoscopic, or robotic BCE. Because a deep, narrow pelvis can hamper MIS distal ureteral dissection, surgeons often adopt a hybrid technique. Consequently, IVR did not differ between groups; however, MIS—including laparoscopic BCE (15.8%) and robotic BCE (11.4%)—was associated with fewer systemic recurrences. The likely advantage stems from superior visualization and precise manipulation, permitting complete bladder-cuff resection and reducing residual ureteral stump risk [13]. Data from 17 centers (185 robotic and 91 laparoscopic cases) showed a higher likelihood of adequate BCE with robotic surgery [14]. MIS affords unobstructed access to the distal ureter, enabling clear identification of Waldeyer’s sheath, meticulous intramural ureter dissection, watertight cystorrhaphy, and avoidance of urine spillage.

With increased experience in distal ureterectomy and BCE—via open or MIS approaches—surgeons usually encounter two key tubular structures at the distal ureter: (i) the medial umbilical ligament, which crosses anterior to the ureter below its midpoint [15]; and (ii) the superior vesical artery, which traverses the ureterovesical junction. Ligation and division of the superior vesical artery allow caudal ureteral traction, exposing the fibromuscular Waldeyer’s sheath around the intramural segment. The detrusor muscle at the ureteral hiatus can then be sharply divided. This *tenting maneuver* permits en bloc removal of the intramural ureter and minimizes the risk of a residual stump.

Although Kapoor et al. [16] and Tsuboi et al. [17] reported better IVR-free survival with transvesical versus extravesical BCE, the extravesical technique is increasingly favored in MIS RNU because it shortens operative time. It also avoids cystotomy closure and reduces the risk of tumor-bearing urine spillage. However, inaccurate identification of the distal ureter during extravesical BCE can compromise oncologic control; the magnified, three-dimensional view of MIS, combined with careful attention to anatomic landmarks, mitigates this risk.

This study has several limitations. First, not all patients in the MIS group underwent laparoscopic or robotic intracorporeal BCE. However, our cohort reflects real-world practices in managing UTUC, where patient factors such as obesity or a deep and narrow pelvis can hinder adequate exposure of the distal ureter, necessitating open BCE for proper oncological control. Second, detailed information regarding the use of transvesical or extravesical BCE and the presence of residual ureteral stumps could not be comprehensively assessed due to the multicenter retrospective nature of the study. Variations existed in the BCE technique, and this potential confounder could not be incorporated into the multivariate or propensity-score models. Finally, the MIS approach did not demonstrate a significant advantage in reducing postoperative morbidities. This finding may be attributable to the learning curve and the varying levels of surgical experience across institutions.

## Conclusion

This study demonstrates that MIS approaches, including laparoscopic and robotic RNU, may offer superior survival outcomes to open RNU for patients with UTUC. Despite a higher prevalence of adverse pathological features in the MIS cohort, propensity-score-matched analysis revealed significantly improved survival outcomes.

## Electronic supplementary material

Below is the link to the electronic supplementary material.

Supplementary Material 1

Supplementary Material 2

## Data Availability

No datasets were generated or analysed during the current study.

## References

[1] Chen CY, Chang CH, Yang CR, Hsieh KL, Tsing WH, Chen IA et al (2024) Prognostic factors of intravesical recurrence after radical nephroureterectomy for upper tract urothelial carcinoma. World J Urol 42:22 38197890 10.1007/s00345-023-04700-9

[2] Clayman RV, Kavoussi LR, Figenshau RS, Chandhoke PS, Albala DM (1991) Laparoscopic nephroureterectomy: initial clinical case report. J Laparoendosc Surg 1:343–349 1838941 10.1089/lps.1991.1.343

[3] Rose K, Khan S, Godbole H, Olsburgh J, Dasgupta P, Guy S et al (2006) Robotic assisted retroperitoneoscopic nephroureterectomy -- first experience and the hybrid Port technique. Int J Clin Pract 60:12–14 16409422 10.1111/j.1368-5031.2006.00703.x

[4] Trecarten S, Bhandari M, Abdelaziz A, Noel O, Liss M, Dursun F et al (2024) Open versus minimally invasive nephroureterectomy in octogenarians: an analysis of surgical approach trends, outcomes, and survival analysis with propensity matching. Urol Oncol 42:220 e9- e19 10.1016/j.urolonc.2024.02.005 38631967

[5] Peyronnet B, Seisen T, Dominguez-Escrig JL, Bruins HM, Yuan CY, Lam T et al (2019) Oncological outcomes of laparoscopic nephroureterectomy versus open radical nephroureterectomy for upper tract urothelial carcinoma: an European association of urology guidelines systematic review. Eur Urol Focus 5:205–223 29154042 10.1016/j.euf.2017.10.003

[6] Piszczek R, Nowak L, Krajewski W, Chorbinska J, Poletajew S, Moschini M et al (2021) Oncological outcomes of laparoscopic versus open nephroureterectomy for the treatment of upper tract urothelial carcinoma: an updated meta-analysis. World J Surg Oncol 19:129 33882936 10.1186/s12957-021-02236-z PMC8061074

[7] Chen IA, Chang CH, Huang CP, Wu WJ, Li CC, Chen CH et al (2021) Factors predicting oncological outcomes of radical nephroureterectomy for upper tract urothelial carcinoma in Taiwan. Front Oncol 11:766576 35096575 10.3389/fonc.2021.766576 PMC8793058

[8] Colin P, Ouzzane A, Yates DR, Audenet F, Pignot G, Arvin-Berod A et al (2012) Influence of positive surgical margin status after radical nephroureterectomy on upper urinary tract urothelial carcinoma survival. Ann Surg Oncol 19:3613–3620 22843187 10.1245/s10434-012-2453-9

[9] Ditonno F, Franco A, Wu Z, Wang L, Abdollah F, Simone G et al (2024) Robot-assisted nephroureterectomy: surgical and mid-term oncological outcomes in over 1100 patients (ROBUUST 2.0 collaborative group). BJU Int 134(6):967–975 10.1111/bju.16526 39263834

[10] Rajan K, Khalifa A, Geraghty R, Parmar K, KandaSwamy G, Gomez Rivas J et al (2023) Oncological efficacy of robotic nephroureterectomy vs. open and laparoscopic nephroureterectomy for suspected non-metastatic UTUC—a systematic review and meta-analysis. Cancers (Basel) 15(20):4926 10.3390/cancers15204926 PMC10605607 37894293

[11] Veccia A, Antonelli A, Francavilla S, Simeone C, Guruli G, Zargar H et al (2020) Robotic versus other nephroureterectomy techniques: a systematic review and meta-analysis of over 87,000 cases. World J Urol 38:845–852 31773242 10.1007/s00345-019-03020-1

[12] Chang CL, Tsai CY, Cheng PY, Wu WJ, Tsai YC (2025) Robot-assisted radical nephroureterectomy: a safe and effective option for upper tract urothelial carcinoma, especially for novice surgeons. Cancers (Basel) 17(9):1394 10.3390/cancers17091394 PMC12071112 40361320

[13] Pathak RA, Hemal AK (2020) Fate of residual ureteral stump in patients undergoing robot-assisted radical nephroureterectomy for high-risk upper tract urothelial carcinoma. Transl Androl Urol 9:856–862 32420200 10.21037/tau.2019.09.14 PMC7214963

[14] Veccia A, Carbonara U, Djaladat H, Mehazin R, Eun DD, Reese AC et al (2022) Robotic vs laparoscopic nephroureterectomy for upper tract urothelial carcinoma: a multicenter propensity-score matched pair tetrafecta analysis (ROBUUST collaborative Group). J Endourol 36:752–759 35019760 10.1089/end.2021.0587

[15] Wu Z, Li M, Wang J, Veccia A, Xu Y, Zhang C et al (2021) Pure retroperitoneoscopic extravesical standardized seeable (PRESS) excision of distal ureter and bladder cuff in radical nephroureterectomy: step-by-step technique. Minerva Urol Nephrol 73:392–400 32284528 10.23736/S2724-6051.20.03711-X

[16] Kapoor A, Dason S, Allard CB, Shayegan B, Lacombe L, Rendon R et al (2014) The impact of method of distal ureter management during radical nephroureterectomy on tumour recurrence. Can Urol Assoc J 8:E845–E852 25485014 10.5489/cuaj.1985 PMC4250251

[17] Tsuboi I, Matsukawa A, Kardoust Parizi M, Klemm J, Schulz RJ, Cadenar A et al (2024) Differential effect of surgical technique on intravesical recurrence after radical nephroureterectomy in patients with upper tract urothelial cancer: a systematic review and Meta-analysis. World J Urol 42:488 39162743 10.1007/s00345-024-05185-w PMC11335797

