# Supplementary material for: Comparative survival outcomes of minimally invasive versus open radical nephroureterectomy for upper tract urothelial carcinoma in Taiwan
Source: World J Urol. 2025 Jul 30;43(1):463. doi: 10.1007/s00345-025-05829-5 (PMC12310803; doi:10.1007/s00345-025-05829-5)
Supplement: Supplementary file 1 — Supplementary Material 1 [file 345_2025_5829_MOESM1_ESM.docx]

| Univariate analysis | OS | | CSS | | DFS | |
| --- | --- | --- | --- | --- | --- | --- |
|  | HR (95% CI) | p-value | HR (95% CI) | p-value | HR (95% CI) | p-value |
| Group | |  |  |  |  |  |
| Open | 1 |  | 1 |  | 1 |  |
| MIS | 0.635 (0.538, 0.750) | <0.001** | 0.727 (0.572, 0.924) | 0.009** | 0.623 (0.513, 0.758) | <0.001** |
| Sex |  |  |  |  |  |  |
| Male | 1 |  | 1 |  | 1 |  |
| Female | 0.853 (0.728, 0.998) | 0.048* | 0.966 (0.762, 1.224) | 0.772 | 0.916 (0.756, 1.110) | 0.371 |
| Age |  |  |  |  |  |  |
| <70 | 1 |  | 1 |  | 1 |  |
| >=70 | 2.405 (2.045, 2.829) | <0.001** | 2.141 (1.688, 2.717) | <0.001** | 1.577 (1.301, 1.911) | <0.001** |
| Laterality |  |  |  |  |  |  |
| Left | 1 |  | 1 |  | 1 |  |
| Right | 1.026 (0.877, 1.202) | 0.747 | 0.903 (0.713, 1.143) | 0.396 | 0.857 (0.707, 1.038) | 0.115 |
| Tumor location |  |  |  |  |  |  |
| Renal pelvis | 1 |  | 1 |  | 1 |  |
| Ureter | 1.339 (1.120, 1.599) | 0.001** | 1.549 (1.179, 2.036) | 0.002** | 1.431 (1.147, 1.784) | 0.001** |
| Renal pelvis + Ureter | 1.651 (1.334, 2.042) | <0.001** | 2.053 (1.504, 2.802) | <0.001** | 1.870 (1.454, 2.405) | <0.001** |
| Cell Type |  |  |  |  |  |  |
| urothelial | 1 |  | 1 |  | 1 |  |
| UC with variants | 1.222 (0.889, 1.679) | 0.216 | 1.736 (1.173, 2.568) | 0.006** | 1.272 (0.904, 1.791) | 0.168 |
| others | 2.806 (1.254, 6.280) | 0.012* | 4.709 (1.940, 11.433) | 0.001** | 2.854 (1.273, 6.401) | 0.011* |
| Multifocality |  |  |  |  |  |  |
| No | 1 |  | 1 |  | 1 |  |
| Yes | 1.611 (1.371, 1.894) | <0.001** | 1.953 (1.539, 2.477) | <0.001** | 1.786 (1.472, 2.166) | <0.001** |
| RNU histology |  |  |  |  |  |  |
| low grade | 1 |  | 1 |  | 1 |  |
| high grade | 1.949 (1.430, 2.656) | <0.001** | 5.370 (2.655, 10.862) | <0.001** | 5.075 (2.919, 8.825) | <0.001** |
| G2 | 2.127 (1.487, 3.042) | <0.001** | 3.079 (1.361, 6.966) | 0.007** | 4.557 (2.364, 8.784) | <0.001** |
| Urinary bladder tumor |  |  |  |  |  |  |
| No | 1 |  | 1 |  | 1 |  |
| Previous Hx of bladder UC | 1.420 (1.044, 1.932) | 0.026* | 0.875 (0.509, 1.505) | 0.630 | 1.202 (0.815, 1.772) | 0.353 |
| Concurrent Bladder UC | 1.768 (1.465, 2.132) | <0.001** | 1.612 (1.219, 2.132) | 0.001** | 1.553 (1.236, 1.951) | <0.001** |
| CIS |  |  |  |  |  |  |
| No | 1 |  | 1 |  | 1 |  |
| Yes | 0.872 (0.708, 1.073) | 0.195 | 0.983 (0.737, 1.312) | 0.908 | 0.828 (0.653, 1.051) | 0.122 |
| Lymphovascular invasion |  |  |  |  |  |  |
| No | 1 |  | 1 |  | 1 |  |
| Yes | 1.976 (1.623, 2.404) | <0.001** | 2.856 (2.210, 3.690) | <0.001** | 2.306 (1.866, 2.851) | <0.001** |
| Surgical margin |  |  |  |  |  |  |
| Free | 1 |  | 1 |  | 1 |  |
| Positive | 4.395 (3.166, 6.101) | <0.001** | 7.064 (4.894, 10.198) | <0.001** | 5.343 (3.861, 7.395) | <0.001** |
| Tumor Necrosis |  |  |  |  |  |  |
| No | 1 |  | 1 |  | 1 |  |
| Yes | 1.485 (1.183, 1.865) | 0.001** | 2.075 (1.552, 2.773) | <0.001** | 1.672 (1.313, 2.129) | <0.001** |
| Pathological stage T |  |  |  |  |  |  |
| pTis/pTa/pT0/pT1 | 1 |  | 1 |  | 1 |  |
| pT2 | 1.408 (1.136, 1.744) | 0.002** | 2.969 (1.985, 4.439) | <0.001** | 2.347 (1.740, 3.164) | <0.001** |
| pT3 | 2.100 (1.743, 2.532) | <0.001** | 7.142 (5.085, 10.032) | <0.001** | 4.734 (3.682, 6.086) | <0.001** |
| pT4 | 4.455 (3.170, 6.261) | <0.001** | 17.174 (10.713, 27.531) | <0.001** | 10.884 (7.314, 16.197) | <0.001** |
| Adjuvant chemotherapy |  |  |  |  |  |  |
| No | 1 |  | 1 |  | 1 |  |
| Yes | 1.053 (0.852, 1.301) | 0.633 | 1.617 (1.228, 2.130) | 0.001* | 1.413 (1.127, 1.772) | 0.003** |
| Adjuvant radiation therapy |  |  |  |  |  |  |
| No | 1 |  | 1 |  | 1 |  |
| Yes | 1.709 (1.177, 2.482) | 0.005** | 2.494 (1.546, 4.022) | <0.001** | 2.477 (1.676, 3.659) | <0.001** |
| RNU date |  |  |  |  |  |  |
| Before the end of 2010 | 1 |  | 1 |  | 1 |  |
| After the start of 2011 | 0.726 (0.608, 0.867) | <0.001** | 0.816 (0.631, 1.056) | 0.123 | 0.762 (0.612, 0.948) | 0.015* |
| CAD |  |  |  |  |  |  |
| No | 1 |  | 1 |  | 1 |  |
| Yes | 1.233 (0.859, 1.771) | 0.256 | 1.142 (0.667, 1.955) | 0.629 | 0.873 (0.550, 1.385) | 0.565 |
| HTN |  |  |  |  |  |  |
| No | 1 |  | 1 |  | 1 |  |
| Yes | 1.279 (1.085, 1.506) | 0.003** | 1.166 (0.918, 1.481) | 0.208 | 1.147 (0.946, 1.390) | 0.164 |
| ESRD (HD PD) |  |  |  |  |  |  |
| No | 1 |  | 1 |  | 1 |  |
| Yes | 1.047 (0.818, 1.341) | 0.713 | 0.547 (0.347, 0.863) | 0.009** | 0.697 (0.506, 0.958) | 0.026 |
| DM |  |  |  |  |  |  |
| No | 1 |  | 1 |  | 1 |  |
| Yes | 1.509 (1.257, 1.811) | <0.001** | 1.315 (1.001, 1.728) | 0.049* | 1.198 (0.959, 1.496) | 0.112 |
| Malignancy (not UTUC/ bladder UC) |  |  |  |  |  |  |
| No | 1 |  | 1 |  | 1 |  |
| Yes | 1.628 (1.293, 2.052) | <0.001** | 1.483 (1.060, 2.076) | 0.022 | 0.979 (0.725, 1.323) | 0.890 |

**Supplementary Table 1** Comparative univariate survival analysis the UTUC patients.

Cl, confidence; HR, hazard ratio; OS, overall survival; CSS, cancer-specific survival; DFS, disease-free survival. * < 0.05, ** < 0.01.
