# Supplementary material for: Comparative survival outcomes of minimally invasive versus open radical nephroureterectomy for upper tract urothelial carcinoma in Taiwan
Source: World J Urol. 2025 Jul 30;43(1):463. doi: 10.1007/s00345-025-05829-5 (PMC12310803; doi:10.1007/s00345-025-05829-5)
Supplement: Supplementary file 2 — Supplementary Material 2 [file 345_2025_5829_MOESM2_ESM.docx]

**Supplementary Table 2** Comparative multivariate survival analysis the UTUC patients.

| Multivariate analysis | OS | | CSS | | DFS | |
| --- | --- | --- | --- | --- | --- | --- |
|  | HR (95% CI) | p-value | HR (95% CI) | p-value | HR (95% CI) | p-value |
| Group | |  |  |  |  |  |
| Open | 1 |  | 1 |  | 1 |  |
| MIS | 0.662 (0.546, 0.802) | <0.001** | 0.659 (0.506, 0.859) | 0.002** | 0.646 (0.520, 0.802) | <0.001** |
| Sex |  |  |  |  |  |  |
| Male | 1 |  |  |  |  |  |
| Female | 0.807 (0.683, 0.954) | 0.012* |  |  |  |  |
| Age |  |  |  |  |  |  |
| <70 | 1 |  | 1 |  | 1 |  |
| >=70 | 2.441 (2.050, 2.907) | <0.001** | 1.779 (1.370, 2.311) | <0.001** | 1.314 (1.063, 1.624) | 0.012* |
| Tumor location |  |  |  |  |  |  |
| Renal pelvis | 1 |  | 1 |  | 1 |  |
| Ureter | 1.355 (1.114, 1.647) | 0.002** | 1.520 (1.119, 2.065) | 0.007* | 1.389 (1.085, 1.779) | 0.009** |
| Renal pelvis + Ureter | 1.211 (0.902, 1.624) | 0.202 | 1.241 (0.812, 1.897) | 0.318 | 1.185 (0.844, 1.666) | 0.327 |
| Cell Type |  |  |  |  |  |  |
| urothelial | 1 |  | 1 |  | 1 |  |
| UC with variants | 1.091 (0.777, 1.533) | 0.613 | 1.146 (0.749, 1.754) | 0.531 | 0.936 (0.650, 1.348) | 0.722 |
| others | 1.616 (0.594, 4.398) | 0.347 | 2.912 (1.070, 7.926) | 0.036 | 1.723 (0.705, 4.212) | 0.233 |
| Multifocality |  |  |  |  |  |  |
| No | 1 |  | 1 |  | 1 |  |
| Yes | 1.257 (0.997, 1.584) | 0.053 | 1.535 (1.103, 2.137) | 0.011* | 1.530 (1.169, 2.003) | 0.002** |
| RNU histology |  |  |  |  |  |  |
| low grade | 1 |  | 1 |  | 1 |  |
| high grade | 1.364 (0.983, 1.892) | 0.063 | 2.040 (0.985, 4.225) | 0.055 | 2.449 (1.383, 4.336) | 0.002** |
| G2 | 1.548 (1.060, 2.261) | 0.024* | 1.905 (0.811, 4.473) | 0.139 | 2.649 (1.319, 5.323) | 0.006** |
| Urinary bladder tumor |  |  |  |  |  |  |
| No | 1 |  | 1 |  | 1 |  |
| Previous history of bladder UC | 1.517 (1.101, 2.092) | 0.011* | 0.945 (0.532, 1.679) | 0.847 | 1.167 (0.760, 1.790) | 0.480 |
| Concurrent Bladder UC | 1.798 (1.457, 2.219) | <0.001** | 1.596 (1.172, 2.173) | 0.003** | 1.532 (1.191, 1.970) | 0.001** |
| Lymphovascular invasion |  |  |  |  |  |  |
| No | 1 |  | 1 |  | 1 |  |
| Yes | 1.286 (1.020, 1.619) | 0.033* | 1.282 (0.958, 1.716) | 0.095 | 1.108 (0.869, 1.413) | 0.408 |
| Surgical margin |  |  |  |  |  |  |
| Free | 1 |  | 1 |  | 1 |  |
| Positive | 2.180 (1.487, 3.196) | <0.001** | 2.051 (1.313, 3.204) | 0.002** | 2.039 (1.389, 2.993) | <0.001** |
| Tumor Necrosis |  |  |  |  |  |  |
| No | 1 |  | 1 |  | 1 |  |
| Yes | 1.449 (1.137, 1.845) | 0.003** | 1.705 (1.244, 2.338) | 0.001** | 1.497 (1.157, 1.937) | 0.002** |
| Pathological stage T |  |  |  |  |  |  |
| pTis/pTa/pT0/pT1 | 1 |  | 1 |  | 1 |  |
| pT2 | 1.263 (1.004, 1.590) | 0.046* | 2.315 (1.502, 3.569) | <0.001** | 1.987 (1.436, 2.747) | <0.001** |
| pT3 | 1.745 (1.392, 2.187) | <0.001** | 5.691 (3.813, 8.495) | <0.001** | 4.505 (3.325, 6.103) | <0.001** |
| pT4 | 3.839 (2.616, 5.632) | <0.001** | 14.025 (8.118, 24.231) | <0.001** | 10.610 (6.695, 16.814) | <0.001** |
| Adjuvant chemotherapy |  |  |  |  |  |  |
| No |  |  | 1 |  | 1 |  |
| Yes |  |  | 0.645 (0.469, 0.886) | 0.007** | 0.604 (0.462, 0.788) | <0.001** |
| Adjuvant radiation therapy |  |  |  |  |  |  |
| No | 1 |  | 1 |  | 1 |  |
| Yes | 1.251 (0.827, 1.893) | 0.288 | 1.553 (0.909, 2.651) | 0.107 | 1.432 (0.926, 2.214) | 0.107 |
| RNU date |  |  |  |  |  |  |
| Before the end of 2010 | 1 |  |  |  | 1 |  |
| After the start of 2011 | 0.772 (0.612, 0.975) | 0.030* |  |  | 0.870 (0.669, 1.131) | 0.298 |
| HTN |  |  |  |  |  |  |
| No | 1 |  |  |  |  |  |
| Yes | 1.205 (0.996, 1.458) | 0.056 |  |  |  |  |
| ESRD (HD /PD) |  |  |  |  |  |  |
| No |  |  | 1 |  | 1 |  |
| Yes |  |  | 0.622 (0.373, 1.035) | 0.068 | 0.694 (0.487, 0.989) | 0.043* |
| DM |  |  |  |  |  |  |
| No | 1 |  | 1 |  |  |  |
| Yes | 1.255 (1.024, 1.540) | 0.029* | 1.061 (0.790, 1.426) | 0.693 |  |  |
| Malignancy (not UTUC/ bladder UC) |  |  |  |  |  |  |
| No | 1 |  |  |  |  |  |
| Yes | 1.502 (1.167, 1.933) | 0.002** |  |  |  |  |

Cl, confidence; HR, hazard ratio; OS, overall survival; CSS, cancer-specific survival; DFS, disease-free survival. * < 0.05, ** < 0.01
